# The tumor cell‐derived matrix of lobular breast cancer: a new vulnerability

**DOI:** 10.15252/emmm.202013807

**Published:** 2021-02-22

**Authors:** Katelyn J Kozma, Susan J Done, Sean E Egan

**Affiliations:** ^1^ Program in Cell Biology The Peter Gilgan Center for Research and Learning The Hospital for Sick Children Toronto ON Canada; ^2^ Department of Molecular Genetics University of Toronto Toronto ON Canada; ^3^ Department of Laboratory Medicine and Pathobiology Department of Medical Biophysics University of Toronto Toronto ON Canada; ^4^ Laboratory Medicine Program University Health Network Toronto ON Canada

**Keywords:** Cancer

## Abstract

Invasive lobular carcinoma (ILC) of the breast is a very common disease. Despite its prevalence, these tumors are relatively understudied. One reason for this is a relative lack of models for ILC. This challenge was addressed by Brisken and colleagues through development of an intraductal injection‐based xenograft system for the study of ERα^+^ breast cancers, including both ILC and more common invasive ductal carcinoma (IDC; Sflomos *et al*, 2016). In this issue of *EMBO Molecular Medicine,* the same group have applied intraductal injection‐based xenografts to identify novel tumor cell‐specific transcriptional signatures in ILC (Sflomos *et al*, 2021). In doing so they found overexpression of lysyl oxidase‐like 1 (LOXL1) to be both responsible for the frequently seen stiff collagen‐rich extracellular matrix of lobular breast cancer and essential for their robust growth and metastatic dissemination *in vivo*, thereby identifying a novel therapeutic target.

## Graphical abstract

Despite its prevalence, invasive lobular carcinoma (ILC) is relatively understudied. In their recent study, Brisken and colleagues apply intraductal injection based xenografts to characterize tumor‐cell specific transcriptional signatures in ILC and identify a novel therapeutic target.

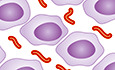

Invasive lobular carcinoma (ILC) of the breast is the most common “special type”, accounting for approximately 10% of all breast tumors. On the basis of 5‐year survival, a typical metric for cancer, ILC, is a relatively good‐prognosis tumor. Indeed, these tumors are mostly estrogen receptor alpha (ERα) positive, express the luminal A molecular signature, and are slow growing—all good prognostic features. Despite this, many patients succumb to recurrent, drug resistant, and metastatic disease years after initial diagnosis and treatment.

Recently, there has been a great deal of excitement over development of specialized assays to grow human tumors in tissues of immunodeficient mice which most closely resemble the tumor’s original source. These specialized xenograft assays have enabled functional characterization of stem‐like cells in cancers, including breast cancer (Al‐Hajj *et al*, 2003). Unfortunately, mammary fat pad xenografts from ERα^+^ breast cancer cell lines or tumors do not always recapitulate histological features or growth kinetics as seen in their source lesion. Indeed, many xenograft tumors transdifferentiate into more basal‐like tumors, which no longer express ERα (Sflomos *et al*, 2016). In 2016, the MIND (mouse intraductal (injection) model) system was adapted to establish xenografts from breast cancer cell lines and tumors within the milk ducts of immunodeficient mice (Behbod *et al*, 2009; Sflomos *et al*, 2016). This approach enabled outgrowth of ERα^+^ tumors, each of which resembled its original source breast cancer. Such lesions took root as carcinoma *in situ*, but progressed, breaking through the basement membrane to invade surrounding mammary tissue and even metastasizing to secondary tissues including bone, lung, and brain. Many MIND xenografts showed excellent response to endocrine therapy.

In their new study, the same authors have used the MIND approach to study ILC (see Fig 1). Remarkably, following intraductal injection of lobular breast cancer cell lines, MDA‐MB‐134‐VI and SUM‐44, lesions formed at, and within, ductal tips. These lobular xenograft structures were reminiscent of grape‐like terminal ductal lobular units (TDLU), seen in the human breast but not in the mouse mammary gland. These outgrowths also contrasted with lesions that formed following injection of ERα‐positive IDC lines (MCF7 or T47D), which grew along the length of ducts, expanding their girth. By histology, the lobular cell lines formed lobular carcinoma in situ (LCIS)‐like lesions, which grew slowly, and ultimately progressed to ILC which metastasized to host adrenal glands, the gastrointestinal tract, ovaries, and/or peritoneal cavity. This pattern of metastatic dissemination is similar to that seen in patients with advanced ILC and quite distinct from patients with advanced IDC as well as in intraductal xenografts of IDC as noted above. As expected, tumors that formed following intraductal injection of lobular cell lines showed the single‐file invasive growth pattern, characteristic of this breast cancer special type.

**Figure 1:**
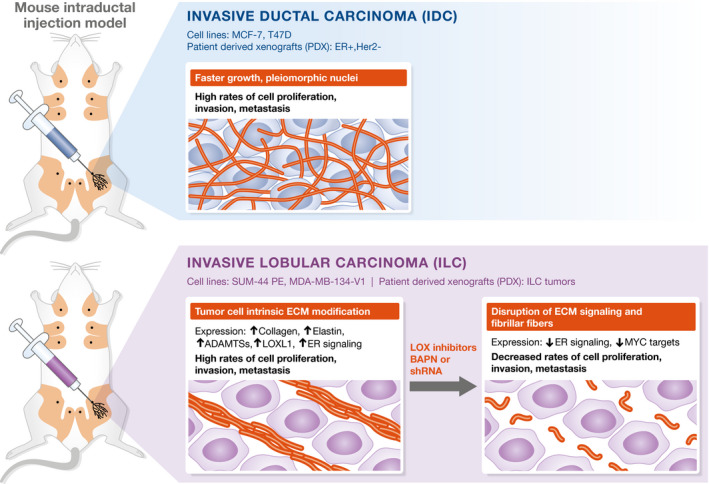
Sflomos *et al* have applied the mouse intraductal injection (MIND) system to study invasive lobular breast cancer (ILC; Sflomos *et al*, 2021) To establish intraductal xenografts, the authors injected ILC cell lines (SUM‐44PE, MDA‐MB‐134‐V1) and cells isolated from patient tumors (patient‐derived xenografts, PDX) into mammary ducts of immunodeficient mice. These lesions were compared to intraductal xenografts established from invasive ductal carcinoma (IDC) cell lines (MCF‐7, T47D) and patient tumors (ER^+^, Her2^−^). Upon engraftment in the mouse, ILC xenografts recapitulate growth patterns, histology, estrogen receptor (ERα) status, and dissemination patterns of human disease. ILC tumors grew in single‐file lines with dense collagen depositions. RNA sequencing indicates that ECM modification is tumor cell intrinsic and unique to ILC tumors. Lobular tumors exhibited increased expression of collagen, Elastin, ADAMTSs, ER signaling, and lysyl oxidase‐like 1 (LOXL1). Lox inhibitors (BAPN) had no effect on IDC tumors but effectively inhibited proliferation and metastasis of ILC intraductal xenografts.

Serial transplantation was used to expand intraductal xenografts, which were in turn used to purify lobular vs ductal tumor cell populations, each free from stroma. RNA sequencing was then used to identify gene signatures associated with each. Remarkably, genes involved in extracellular matrix production and organization were highly enriched in RNA from lobular tumor cells. This ECM remodeling signature included collagen genes, elastin, laminins, and proteases, and could effectively distinguish lobular from ductal breast cancer samples. These data are consistent with high‐level accumulation of intratumoral collagen seen in many ILC (Natal *et al*, 2019). Next, Sflomos *et al* found high‐level expression of lysyl oxidase‐like 1 (LOXL1) in lobular but not ductal breast tumor cells. This prompted them to test for therapeutic effects of the broad‐spectrum lysyl oxidase inhibitor β‐aminopropionitrile (BAPN). Indeed, this drug could effectively delay lobular tumor onset when given to mice shortly after tumor cell injection. Also, when given following tumor establishment, it was able to slow tumor progression and decrease metastatic burden in most cases, both by decreasing tumor cell proliferation and increasing apoptosis. BAPN had no effect on intraductal xenografts that grew following injection of T47D IDC cells. Sflomos *et al* went on to show that BAPN could inhibit the outgrowth and progression of patient‐derived lobular breast cancer but not from patient‐derived ductal breast cancers following intraductal xenografts of tumor cell suspensions. Therapeutic response was associated with disruption of the intratumor collagen fiber network in lobular xenografts and linked to decreased expression of genes associated with cell cycle progression as well as estrogen responsiveness. *LOXL1* knockdown also impaired lobular xenograft growth with a coincident decrease in tumor cell proliferation and loss of collagen fiber organization.

These findings are somewhat reminiscent of the important role played by collagen‐rich ECM in very aggressive ductal tumors, including poor prognosis triple negative and Her2^+^ breast cancers. In this case, dense matrix is also secreted and crosslinked, particularly at the invasive front where high‐level TGFβ signaling is linked to tumor associated macrophage infiltration and immune suppression (Acerbi *et al*, 2015). Interestingly, lysyl oxidase genes are induced in response to hypoxia in triple negative breast tumors, with the resulting dense matrix contributing to therapy resistance (Saatci *et al*, 2020).

Lobular breast cancer patients receive the same basic treatment as patients with IDC. The typical patient can receive surgery, hormone therapy, and potentially chemotherapy and/or radiation therapy. Unfortunately, ILC, while slow growing, are highly invasive, sometimes multifocal, and can recur as lethal (therapy‐resistant) disseminated disease many years after initial diagnosis and treatment. Recent discoveries have revealed a potential opportunity to activate anti‐tumor immune surveillance in some patients with ILC, particularly those with immune‐related ILC (Ciriello *et al*, 2015). Also, deletion of *CDH1* in ILC may well activate dependence on ROS1 tyrosine kinase signaling (Bajrami *et al*, 2018). This has led to the establishment of clinical trials for treatment of advanced ILC, specifically the GELATO trial combing chemotherapy and anti PD‐L1 immune checkpoint blockade (NCT03147040), as well as the ROLO trial which combines drugs targeting the estrogen receptor (Fulvestrant) and ROS1 (Crizotinib; NCT03620643). New findings from Cathrin Brisken and colleagues have added another arrow to the quiver. While broad‐spectrum lysyl oxidase inhibitors like BAPN are too toxic for clinical application, other more specific approaches to target LOXL1 could be developed and combined with any of the above therapies. ILC has never received the attention that it deserves; however, with the advent of intraductal injection as an approach to model this disease, the future is starting to look quite promising.
